# Boosting Knowledge Base Automatically via Few-Shot Relation Classification

**DOI:** 10.3389/fnbot.2020.584192

**Published:** 2020-10-27

**Authors:** Ning Pang, Zhen Tan, Hao Xu, Weidong Xiao

**Affiliations:** Science and Technology on Information Systems Engineering Laboratory, National University of Defense Technology, Changsha, China

**Keywords:** knowledge base, relation classification, few-shot learning, distant supervision, multiple instance learning

## Abstract

Relation classification (RC) aims at extracting structural information, i.e., triplets of two entities with a relation, from free texts, which is pivotal for automatic knowledge base construction. In this paper, we investigate a fully automatic method to train a RC model which facilitates to boost the knowledge base. Traditional RC models cannot extract new relations unseen during training since they define RC as a multiclass classification problem. The recent development of few-shot learning (FSL) provides a feasible way to accommodate to fresh relation types with a handful of examples. However, it requires a moderately large amount of training data to learn a promising few-shot RC model, which consumes expensive human labor. This issue recalls a kind of weak supervision methods, dubbed distant supervision (DS), which can generate the training data automatically. To this end, we propose to investigate the task of *few-shot relation classification under distant supervision*. As DS naturally brings in mislabeled training instances, to alleviate the negative impact, we incorporate various multiple instance learning methods into the classic prototypical networks, which can achieve sentence-level noise reduction. In experiments, we evaluate our proposed model under the standard *N*-way *K*-shot setting of few-shot learning. The experiment results show that our proposal achieves better performance.

## 1. Introduction

Relation Classification (RC) is defined as identifying semantic relations between entity pairs in given plain texts, which is a crucial task in automatic knowledge base (KB) construction (Bollacker et al., 2008). Mainstream works on this task mainly follow supervised learning, where large-scale and high-quality training data is required (Zeng et al., 2014; Gormley et al., 2015).

**Figure 1:**
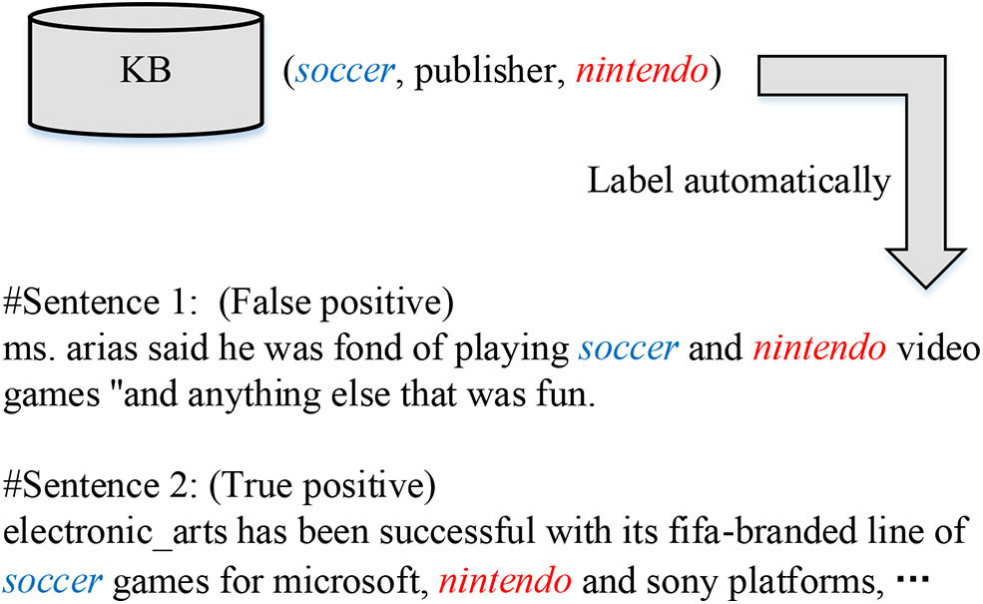
Example of automatic labeling by distant supervision.

However, human-annotated data is always expensive to acquire. Subsequently, recent literature resorted to distant supervision (DS) (Mintz et al., 2009; Riedel et al., 2010) to address the sparsity issue of training data. In DS, it is assumed that *sentences mentioning an entity pair instantiate the relation of the corresponding entity pair in knowledge bases*. With this (untrue) heuristic, large-scale training data can be constructed automatically, but mislabeling is inevitably introduced at the same time. For example, as shown in Figure 1, since the triplet (*soccer*, publisher, *nintendo*) exists in a KB, two sentences mentioning the entity pair (*soccer, nintendo*) are assigned with the relation “publisher.” In fact, the first sentence fails to express the target relation indeed, called false positive instance, while the second one obtains a correct label, which is a true positive instance. Hence, efforts were made to restrain the impact of false positives (Ji et al., 2017; Qin et al., 2018b; Wu et al., 2019). However, these models perform well on common relations, but suffer a dramatic performance drop in classifying long-tail relations, which have few training instances; that is, even though a large amount of training data can be generated by DS, the distributions of such data over different types are unbalanced. Furthermore, they are unable to recognize new relations that have not been seen in training, which potentially restricts their applications in certain scenarios that involve fresh relations in testing.

Lately, pioneering work (Han et al., 2018; Gao et al., 2019) has tried to formulate RC into few-shot learning (FSL) framework (Miller et al., 2000), which aims at accommodating new classes with few examples, while demanding less manual labor than generic supervised learning for fresh relations. Many efforts have made on the few-shot classification task. The early researches fine-tune models which are pre-trained with common classes containing adequate instances by transfer learning (Caruana, 1994; Donahue et al., 2014). After that, metric learning is proposed to project different classes into a distance space (Vinyals et al., 2016; Snell et al., 2017), where similar classes are placed close to each other. Lately, optimization-based meta-learning is developed fast because of its fast-learning ability to learn from previous experience and generalize to new knowledge (Finn et al., 2017; Ravi and Larochelle, 2017). These models, especially prototypical networks, achieve promising results on several benchmarks, but almost all of them focus on image processing. Observing the lack of researches about employing FSL to natural language processing (NLP) tasks, this paper focus on the few-shot relation classification with distant supervision data.

**Figure 2:**
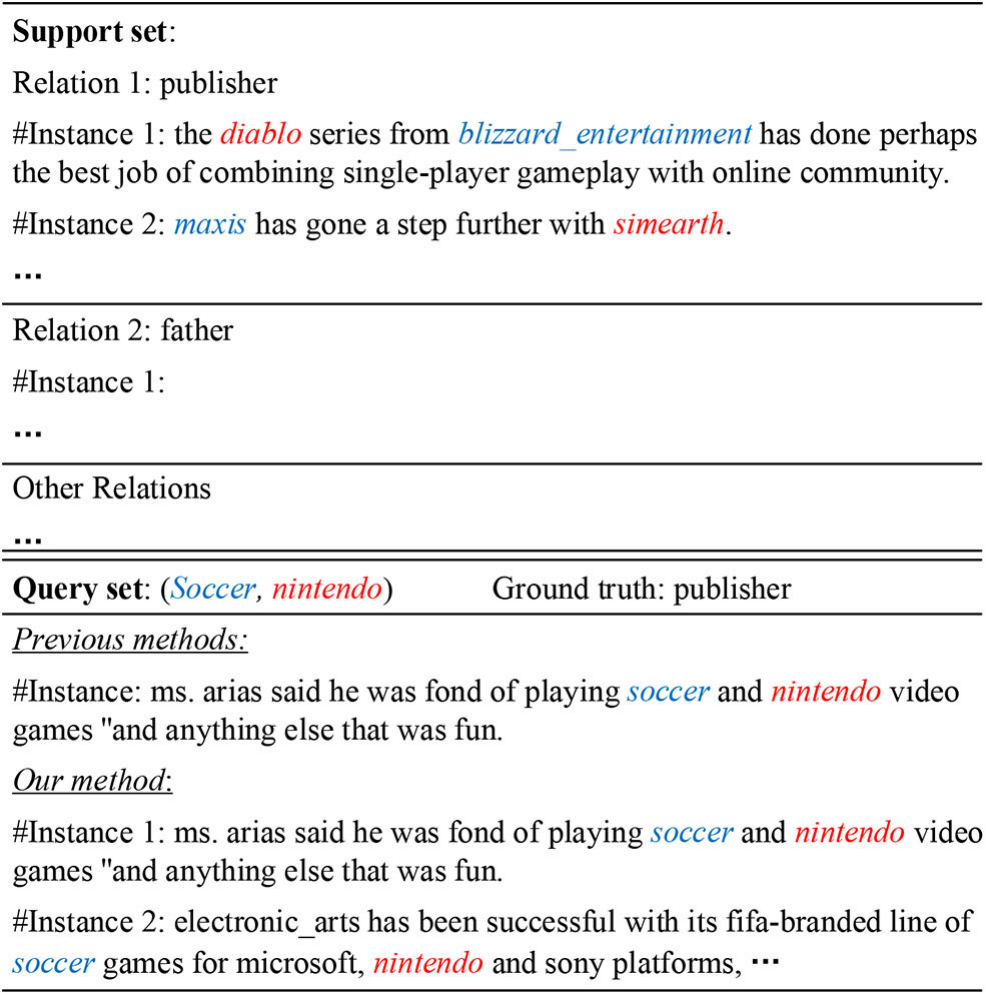
An example of few-shot relation classification. Previous work selects a single instance as the query, which may fail to provide enough information express the semantic relation between the two entities. To solve the problem, we take multiple instances concerning the same entity pair as the query.

Figure 2 shows an example of few-shot relation classification (FSRC). For an unlabeled query, this method is aimed at classifying it into a correct relation based on a few support instances for each relation. Although FSL requires less training examples in predicting a new relation, moderately large-scale labeled data is necessary to train a promising FSRC model. In specific, a dataset was *manually* labeled for FSRC, namely, FewRel and on top of it, systematic evaluation of state-of-the-art FSL methods (used in computer vision) was carried out for RC (Han et al., 2018). Note that FewRel was constructed by crowdsourcing, and thus, a number much larger than 64 × 700 of annotations are necessary, where 64 (700, resp.) is the number of relations (labeled instances each relation, resp.) thereof.

### 1.1. Motivation

To recap, DS can generate large-scale data but suffers from long-tail relations and mislabels; meanwhile, FSRC is able to recognize new relations with few training samples, but requires moderately large amount of human labor for data annotation. Hence, we are at the frontiers of DS and FSRC, being ready to union them, in order to compensate for the downsides of the two paradigms. The combination of DS and FSRC enables the fully automatic method to develop a RC model, which can extract the relation held between two entities. Subsequently, the extracted triplets are used to boost the knowledge base automatically.

In this research, we investigate the task of *few-shot relation classification under distant supervision*. In realization, we refine a previous DS dataset (Zeng et al., 2017), which was built by aligning Wikidata with New York Times corpus, into a reconstructed dataset for FSRC. The DS data is collected automatically, and more details can be seen in Zeng et al. (2017). Taking for granted that the sentences mentioning an entity pair instantiate the relation of the corresponding entities in KBs, DS data is born with *mislabels* (Riedel et al., 2010). From the example shown in Figure 2, we can see that, in the new scenario of distantly supervised FSRC, both support and query sets are practically noisy. If a single false positive instance is sampled as a query like previous studies (Han et al., 2018; Fan et al., 2019; Gao et al., 2019), it cannot be classified into an appropriate relation in the support set. Since a few-shot model is optimized by minimizing the loss of the predictions over the queries, sampling a mislabeled instance as the query will inevitably mislead the optimization process. To tackle this problem, we follow the *at-least-once* assumption, and take an instance bag as a query:

**Definition 1 (At-least-once assumption)**. *If two entities participate in a relation, at-least one sentence mentioning these two entities express the relation*.

**Definition 2 (Instance bag)**. *All sentences mentioning a particular entity pair make an instance bag*.

Based on the at-least-once assumption, an instance bag contains enough semantic information to express the relation between the target entities. Therefore, selecting instance bags as queries alleviates the problem of misleading the optimization process which is caused by mislabeled instances. Besides, to alleviate the impact of false positives in the bag, we resort to *multiple instance learning* (MIL) methods, which assigns a single label to the instance bag, and achieve sentence-level noise reduction.

In previous research on FSRC, prototypical networks (PN) achieve promising performance (Han et al., 2018) by measuring the distances between a query and prototypes. The classic approach (Snell et al., 2017) first encodes all instances into a unified vector space, then generates each prototype by averaging all support instances of a relation type. Nevertheless, the mislabeled support instance sampled from DS data may cause a huge deviation for the prototype. In this connection, we conceive a *attention-based MIL* method, which consists of two steps:

- *Denoising the query set*: as discussed above, selecting a single instance that is unfortunately mislabeled as query has negative effects on the optimization of few-shot models. Thus, we take an instance bag as a query which provides enough information for the few-shot models to recognize an appropriate relation concerning an entity pair. Besides, self-attention is supplied to dynamically denoise while producing a more informative query feature vector;
- *Denoising the support set*: for the instances selected as the support set for each relation, to mitigate the issue of substantial deviation of the learned prototype due to mislabeled support instances, support instance-level attention is leveraged to generate a more representative prototype.

In previous studies, Gao et al. (2019) have investigated the support instance-level attention to strengthen the robustness of PN to the noise in support set. However, our work differs from Gao et al. (2019) in two perspectives: (1) Gao et al. (2019) regard the diversity of text as the noise, while in our research, the noise (i.e., mislabeled instances) in the support set is naturally brought in by distant supervision, which is more challenging to be solved; (2) as mentioned above, Gao et al. (2019) select a single instance as the query, which negatively affects the optimization process of few-shot models when distant supervision data is used for training. Differently, we take an instance bag as the query and employ MIL methods to denoise the instance bag. In addition, to evaluate our model on the task of few-shot relation classification under distant supervision, we reconstructed an DS dataset for FSRC.

### 1.2. Contributions

To sum up, we are among the first to propose to investigate a new task of few-shot relation classification under distant supervision, and the technical contribution is at least three-fold:

- We adapt existing DS data for RC to confront to FSL scenarios, which enables a fully automatic way for FSRC to obtain large-scale potentially-unbiased training data;
- We conceive an attention-based multiple instance learning method over prototypical networks, which reduces noise and emphasizes important signals at both support and query instance levels;
- The proposed task and method are empirically evaluated, and comprehensive results verify the superiority of our proposal over competitors under *N-way* *K-shot* settings.

### 1.3. Organization

In section 2, we formally define the task in this work. Related works are discussed in section 3, then we introduce the methodology in section 4. Afterwards, experimental results and detailed analysis are presented in section 5, followed by conclusion.

## 2. Task Formulation

Formally, the task of *few-shot relation classification under distant supervision* with attention-based MIL is to obtain a function

(1) $$\begin{array}{l} F:\left(R,S,Q\right)\to r, \end{array}$$

given training data *D*, which is labeled by existing knowledge bases under the DS assumption. In specific, *R* = {*r*_1_, …, *r*_*i*_, …, *r*_*m*_} is the *relation set*, 1 ≤ *i* ≤ *m*, *m* = |*R*|, where |·| denotes the cardinality of a set. *D* = {*D*_*r*_1__, …, *D*_*r*_*i*__, …, *D*_*r*_*m*__}, where *D*_*r*_*i*__ is a set of DS-labeled *instance bags* (all) with relation *r*_*i*_. *S* is the *support set*, i.e.,

(2) $$\begin{array}{l} S=\left\{\left(s_{1}^{r_{1}},s_{2}^{r_{1}},\ldots ,s_{n_{1}}^{r_{1}}\right),\ldots ,\left(s_{1}^{r_{m}},s_{2}^{r_{m}},\ldots ,s_{n_{m}}^{r_{m}}\right)\right\}, \end{array}$$

where relation *r*_*i*_ has *n*_*i*_ support instances, each of which is randomly selected from *D*_*r*_*i*__. $Q=\left\{s_{1}^{q},\ldots ,s_{\left|Q\right|}^{q}\right\}$ (|*Q*| ≥ 1) is the *query set*, which is essentially an instance bag concerning an entity pair.

The query set *Q* gives rise to the major difference with respect to the formulation of conventional FSRC in Han et al. (2018), Fan et al. (2019), and Gao et al. (2019). As DS data tends to have mislabeled instances, if the previous formulation is followed, a mislabeled instance is likely to be sampled as the query instance; in this case, a FSRC model may be intermittently confused during training by the mislabeled query instances, as they substantially deviate from real ones. To overcome the limitation, we propose to employ in training an *instance bag*, instances of which concern the same entity pair and are DS-labeled with the same relation *r*, to replace a single query instance; by doing this, we expect that the trained model can recover the relation *r* given its instance bag. Then for testing, the trained model predicts the best relation, where *single or multiple* instances can be supplied.

In this research, we adopt *N-way* *K-shot* setting, which has been widely used in FSRC (Han et al., 2018; Fan et al., 2019; Gao et al., 2019), i.e., *N* = *m*, and *K* = *n*_1_ = ⋯ = *n*_*m*_.

## 3. Related Work

Knowledge base is becoming increasingly important for many downstream applications, and there are various methods to boost the knowledge base (Chen et al., 2019; Zhao et al., 2020). Relation classification (RC) is a vital task for constructing knowledge base automatically. Our work is related to RC via distant supervision (DS) and few-shot learning (FSL). We review the related works as follow.

### 3.1. Relation Classification Under Distant Supervision

Most existing researches concentrate on neural models via supervised learning (Zeng et al., 2014; Nguyen and Grishman, 2015) or distantly supervised learning (Zeng et al., 2015; Lin et al., 2016). Supervised learning requires a large amount of annotated data, which can be fairly expensive to acquire. As a result, many neural models with supervised learning for RC suffer from data insufficiency (Zeng et al., 2014; dos Santos et al., 2015). DS comes as a remedy (Mintz et al., 2009), which can generate large-scale training data without human labor; whereas, it inevitably brings in mislabels and still has little coverage of long-tail relations. Riedel et al. (2010) formulated RC under DS as a *multiple instance learning* problem to alleviate the influence of mislabels, which achieves remarkable improvement.

On the foundation of this work, other feature-based methods (Hoffmann et al., 2011; Surdeanu et al., 2012) are proposed to better handle noise brought in by distant supervision. Besides, representative neural models include (Zeng et al., 2015; Lin et al., 2016; Feng et al., 2018). Among them, Zeng et al. (2015) perform at-least-one multiple instance learning on DS data. To fully exploit information in an instance bag, Lin et al. (2016) proposed selective attention over instances to dynamically remove noisy samples. Lately, reinforcement learning (Feng et al., 2018; Zeng et al., 2018) and generative adversarial network (Qin et al., 2018a) were combined with these models to further alleviate noise. These models define the task relation extraction as a multiclass classification problem, and they can only extract limited relations as a result. Our work is connected to DS, the major difference is that our proposal is formulated under a FSL framework, which can find new relations in testing, and solve the long-tail relation problem.

### 3.2. Relation Classification via Few-Shot Learning

Despite satisfactory performance, the aforementioned models show limitations in handling relations with few training samples. FSL provides a feasible solution to the problem of recognizing new classes, which aims at adapting to new classes, given only a few training samples of these classes. Many efforts are devoted to transfer learning methods, which generalizes to new concepts by fine-tuning models pretrained with common classes containing adequate instances (Caruana, 1994; Bengio, 2012; Donahue et al., 2014). Some model-based meta-learning models achieve the rapid learning by designing a special memory unit (Santoro et al., 2016; Munkhdalai and Yu, 2017; Mishra et al., 2018). Another group of studies focus on optimization-based approaches (Finn et al., 2017; Al-Shedivat et al., 2018), which either generate the model parameters directly or predicting the updating gradients for parameters. Afterwards, metric learning is proposed to project instances into a unified feature space, where instances with the same class are placed adjacent with each other (Koch et al., 2015; Vinyals et al., 2016). Prototypical networks used in Han et al. (2018) and Gao et al. (2019), as well as this research is a representative method of metric learning.

As introduced in section 1, Han et al. (2018) first formulated the task of FSRC, and a dataset FewRel for evaluating the task was created via crowdsourcing. Based on FewRel, Fan et al. presented large-margin prototypical networks with fine-grained features (Fan et al., 2019). Wu et al. proposed a dynamic prototypes selection approach with attention to fully capture information in support set (Wu et al., 2020). Seeing texts are more flexible and noisy than images, Gao et al. (2019) devised tailored prototypical networks, distinguishing itself from those used in the area of computer vision. In particular, based on FewRel, noise was introduced by replacing at a certain probability each support instance with a random instance of different relation labels. In contrast, we address the noise issue, i.e., mislabeled instances in specific, which is naturally brought in when investigating the new task of distantly supervised FSRC, which can be comparatively more challenging.

## 4. Methodology

**Figure 3:**
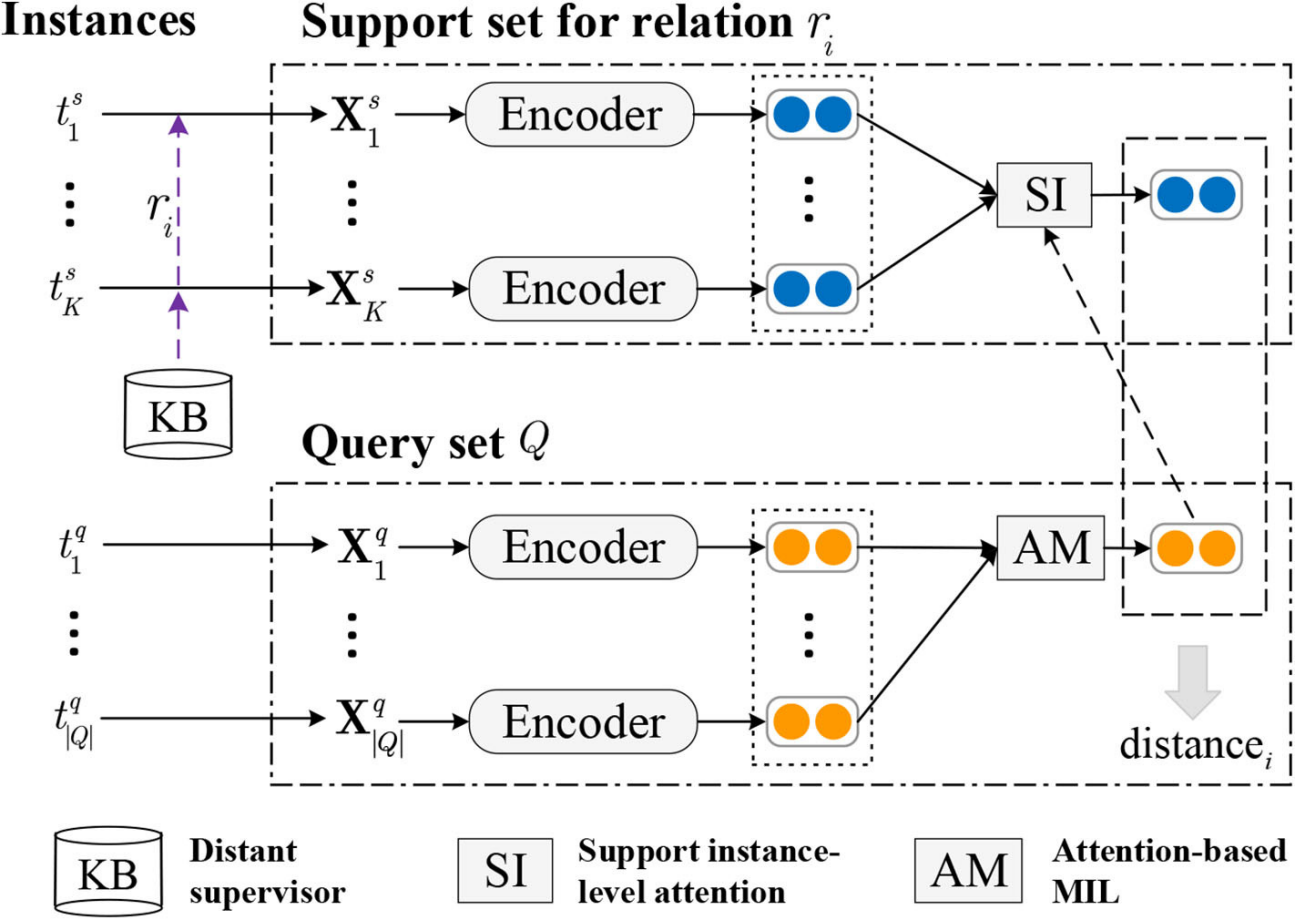
Framework of model for FSRC under DS. *t*^*s*^ and *t*^*q*^ denotes the support instance and query instance respectively, while **X**^*s*^ and **X**^*q*^ are the embedding corresponding representations of *t*^*s*^ and *t*^*q*^.

As shown in Figure 3, the model is established based on prototypical networks (PN), incorporating attention-based multiple instance learning. In this paper, instances are encoded by convolution neural network (CNN) before fed into the PN, and other neural networks can also be employed as encoder.

### 4.1. Sentence Encoder

This module is used to extract semantic features of an instance. Given a sentence *t* = {*w*_1_, *w*_2_, …, *w*_*n*_}, we first transform the raw text into a low-dimensional embedding representation, and then feed it to neural networks to obtain a feature vector.

#### 4.1.1. Embedding Layer

In our method, we map each discrete word token into a low-dimensional vector by looking up a table of pre-trained word embeddings (Pennington et al., 2014). As thus, a word *w*_*i*_ in the sentence *t* is converted into a real-valued embedding ${\text{w}}_{i}\in {\mathbb{R}}^{k_{w}}$, which can express the semantic meaning of *w*_*i*_. Besides, we also incorporate position features, which have been shown to be useful for RC (Zeng et al., 2014). For each word *w*_*i*_, it has two relative distances to the two entities. Two position embedding matrices are initialized randomly and each distance can be transformed into a *k*_*p*_-dimensional vector ${\text{p}}_{i}^{j}\in {\mathbb{R}}^{k_{p}}$, *j* ∈ {1, 2}, by looking them up. Then, we concatenate the word embedding and position features as

(3) $$\begin{array}{l} {\text{x}}_{i}=\left[{\text{w}}_{i}:{\text{p}}_{i}^{1}:{\text{p}}_{i}^{2}\right]\in {\mathbb{R}}^{k_{w}+2k_{p}}. \end{array}$$

When gathering the vector representation of all words together, we obtain the input embedding matrix **X** = {**x**_1_, **x**_2_, …, **x**_*n*_}. After deriving **X**, we feed it into a standard CNN for feature extraction.

#### 4.1.2. Encoding Layer

We use convolution neural network (CNN) as the instance encoder, which is of elegant encoding capability and computing efficiency. **X**_*i*:*j*_ is the concatenation of word vectors [**x**_*i*_:**x**_*i*+1_:⋯:**x**_*j*_]. The weight matrix of the sliding filter with a window size of ω is denoted by $\text{W}\in {\mathbb{R}}^{\omega \times \left(k_{w}+2k_{p}\right)}$. The convolution operation is to take a dot production between **W** and **X**_(*j*−ω+1):*j*_, and generate a vector **c** ∈ ℝ^*m*−ω+1^. Generally, multiple filters are usually required to extract more information, and the corresponding weight matrices are represented by $\hat{\text{W}}=\left\{{\text{W}}_{1},\ldots ,{\text{W}}_{i},\ldots ,{\text{W}}_{d}\right\}$. Each convolution operation can be expressed by

(4) $$\begin{array}{l} c_{ij}={\text{W}}_{i}⊗{\text{X}}_{\left(j-\omega +1\right):j}, \end{array}$$

where *d* is the number of filters, 1 ≤ *i* ≤ *d*, and 1 ≤ *j* ≤ *m* − ω + 1. Afterwards, max-pooling operation is applied on the convolution results to extract the most prominent feature in every dimension, i.e.,

(5) $$\begin{array}{l} y_{i}=\text{ReLU}\left(\underset{1\leq j\leq m}{\max}\left(c_{ij}\right)\right), \end{array}$$

where, ReLU is the activation function in our implementation. Hence, a feature vector for an instance **y**^*i*^ ∈ ℝ^*d*^(*i* ∈ {*s, q*}) is generated by max-pooling layer of CNN, where **y**^*s*^ (resp. **y**^*q*^) denotes support (resp. query) instance.

### 4.2. Attention-Based Multiple Instance Learning Unit

**Figure 4:**
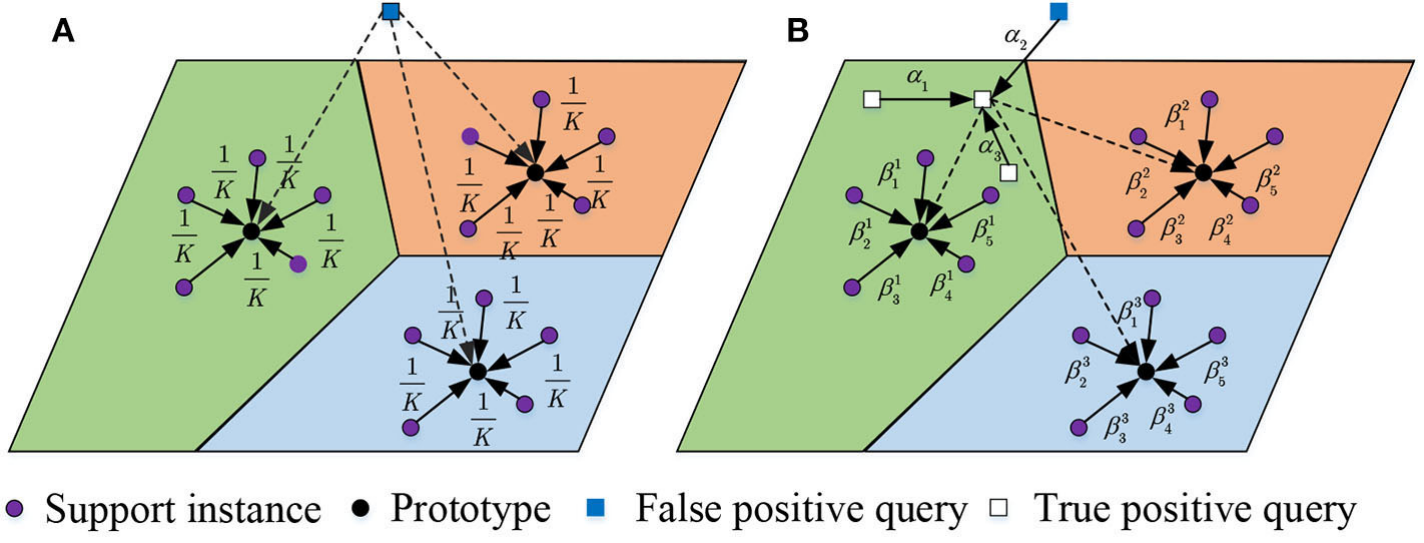
Selection of instances in support and query set. Previous work regards all support instances equally while our method assigns selective attention scores over the support instances. Besides, previous work cannot handle the scenario of false positive queries while our method solves the problem by selecting instance bags as queries. **(A)** Previous method, **(B)** Our method.

Mislabeled instances are harmful to learning and evaluating queries and prototypes. Hence, we conceive an attention-based multiple instance learning unit to mitigate the impact. Figure 4 compares the instance selection of our model in both support and query set with the classic method (Snell et al., 2017). From it, we can see that if a false positive instance is sampled as a query, the previous method cannot handle the situation. Besides, the mean selection of support instances is fixed rather than flexible, which restrains the appropriate selection of support instances given a query.

#### 4.2.1. Attention-Based MIL Pooling in Query Set

Based on multiple instance learning assumption, in the new formulation, it is of necessity to distinguish the instances of various importance. Consequently, given a set of feature vectors for *Q*, namely, $\text{Q}=\left\{{\text{y}}_{1}^{q},\ldots ,{\text{y}}_{\left|Q\right|}^{q}\right\}\in {\mathbb{R}}^{|Q|\times d_{c}}$, our method leverages an attention-based pooling operation over the multiple instances in the bag. We use a self-attention method (Vaswani et al., 2017), which is defined as

(6) $$\begin{array}{l} \text{E}=\text{softmax}\left(\frac{\left({\text{Q}\text{W}}^{1}+{\text{b}}^{1}\right){\left({\text{Q}\text{W}}^{2}+{\text{b}}^{2}\right)}^{⊤}}{\sqrt{d_{c}}}\right), \end{array}$$

where ${\text{W}}^{1},{\text{W}}^{2}\in {\mathbb{R}}^{d_{c}\times d_{c}}$, and ${\text{b}}^{1},{\text{b}}^{2}\in {\mathbb{R}}^{d_{c}}$ are learnable parameters of two linear projection layers, and softmax(·) is the softmax function. **E** ∈ ℝ^|*Q*| × |*Q*|^ is produced by letting each instance attend mutually. And then, we average each row of **E** to generate the attention score for each instance in the query set,

(7) $$\begin{array}{l} \alpha_{i}=\frac{\exp\left(e_{i}\right)}{\overset{\left|Q\right|}{\underset{k=1}{\sum }}\exp\left(e_{k}\right)}, \end{array}$$

(8) $$\begin{array}{l} \;e_{k}=\frac{\overset{\left|Q\right|}{\underset{j=1,j\neq k}{\sum }}{\text{E}}_{kj}}{\left|Q\right|}, \end{array}$$

In this way, the selection of query instances is guided by the high-quality ones in the query set.

Then, the query set representation is obtained by consolidating the feature vectors of query instances in a weighted form, i.e.,

(9) $$\begin{array}{l} {\hat{\text{y}}}^{q}=\overset{\left|Q\right|}{\underset{i=1}{\sum }}\left(\alpha_{i}{\text{y}}_{i}^{q}\right). \end{array}$$

In our implementation, we also try other MIL methods, including the maximum pooling over multiple instance, which is defined as,

(10) $$\begin{array}{l} {\hat{\text{y}}}_{j}^{q}=\underset{1\leq i\leq |Q|}{\max}{\text{Q}}_{ij}. \end{array}$$

We then concatenate all dimensions and obtain the query feature vector ${\hat{\text{y}}}^{q}$.

Another MIL pooling method averages all query instances,

(11) $$\begin{array}{l} {\hat{\text{y}}}^{q}=\frac{1}{\left|Q\right|}\overset{\left|Q\right|}{\underset{i=1}{\sum }}{\text{y}}_{i}^{q}. \end{array}$$

Besides, we also design a perceptron pooling method, which generates the pooling weight for each query instance by,

(12) $$\begin{array}{l} \alpha_{i}=\frac{\exp\left({\text{v}}^{⊤}{\text{y}}_{i}^{q}\right)}{\overset{\left|Q\right|}{\underset{k=1}{\sum }}\exp\left({\text{v}}^{⊤}{\text{y}}_{k}^{q}\right)}, \end{array}$$

where ${\text{v}}^{⊤}\in {\mathbb{R}}^{d_{c}}$ is a parameter vector. The final query vector can be acquired by Equation (9).

#### 4.2.2. Support Instance-Level Attention

Akin to the case of query set, instances in the support set are not equally useful for learning a prototype when a query set is given. Inspired by Gao et al. (2019), we proceed as follows to get a more informative prototype for each relation,

(13) $$\begin{array}{l} {\hat{\text{y}}}^{s}=\overset{K}{\underset{i=1}{\sum }}\left(\beta_{i}{\text{y}}_{i}^{s}\right), \end{array}$$

which is a weighted combination of all support instances, and weight β_*i*_ is calculated according to the *query set*, as the importance of a support instance varies by queries. Thus, β_*i*_ is defined as

(14) $$\begin{array}{l} \beta_{i}=\frac{\exp\left(e_{i}\right)}{\overset{K}{\underset{k=1}{\sum }}\exp\left(e_{k}\right)}, \end{array}$$

(15) $$\begin{array}{l} e_{k}=||\sigma \left({\text{y}}_{k}^{s}{\text{W}}^{s}⊙{\hat{\text{y}}}^{q}{\text{W}}^{s}\right)|{|}_{1}, \end{array}$$

where ||·||_1_ is L1-norm, σ(·) is a hyperbolic tangent function, **W**^*s*^ is a learnable parameter matrix, and **y**^*q*^ is the feature vector of a query generated by Equation (9).

Our proposed attention-based MIL method has two advantages. Firstly, it has *flexibility* in assigning different weights to instances within a query bag, which produces highly informative query vector for bag-level classification. In addition, different attention methods in query set and support set has *interpretability*. High attention weights should be assigned to instances which are true positives, while false positives get low scores.

### 4.3. Prototypical Networks

The basic idea of prototypical networks is to use prototypes, each generated by a support set, respectively, to delegate a relation. Given a query set *Q*, distances between its feature vector ${\hat{\text{y}}}^{q}$ and all the prototypes are calculated, respectively. Then, the entity pair concerned by the query set is classified as *r*_*i*_, if the prototype of *r*_*i*_ is of the smallest distance; the probability of *Q* possessing *r*_*i*_ is

(16) $$\begin{array}{l} p\left(r_{i}|Q\right)=\frac{\text{exp}\left({-||{\hat{\text{y}}}_{i}^{s}-{\hat{\text{y}}}^{q}||}_{2}^{2}\right)}{\overset{m}{\underset{j=1}{\sum }}\text{exp}\left(-||{\hat{\text{y}}}_{j}^{s}-{\hat{\text{y}}}^{q}|{|}_{2}^{2}\right)}. \end{array}$$

To train the model, we use cross-entropy loss as the target,

(17) $$\begin{array}{l} J\left(\Theta \right)=-\underset{j}{\sum }\text{log}p\left(r_{i}|Q_{j};\Theta \right), \end{array}$$

where Θ is the set of parameters used in the model. During model optimization, stochastic gradient decent (SGD) is harnessed to maximize the objective function by updating parameters used in the model iteratively until convergence. For each iteration, mini-batches of samples are selected from the training set.

## 5. Experiments

### 5.1. Data Preparation and Setup

#### 5.1.1. Dataset

To evaluate the proposed task, we constructed a dataset named DS-Few^1^, based on two widely-used DS datasets. The first one was originally built by aligning New York Times corpus with Wikidata^2^. Following the construction method of FewRel, we grouped the instances according to their semantic relations^3^, and obtained the *basic version* of DS-Few, consisting of 87 relations with 192, 142 instances (61, 361 entity pairs) in total^4^. We used 60, 10, and 17 relations for training, validation, and testing, respectively.

For further evaluation, we built an *alternative version* of DS-Few by employing as *test set* another DS dataset—NYT10 (Riedel et al., 2010). Akin to the aforementioned procedure, it was first grouped, and then, we filtered out the sentences that literally appeared in the training set, but retaining the clusters even if the corresponding relations appear in the training set. Eventually, we got a test of 20 relations (10 are seen in the training set) with 142, 424 instances in total. In summary, the two versions represent scenarios that are both possible in real life, i.e., a relation may be seen or not during training.

#### 5.1.2. Parameter Setting

For the initial embedding layer of the sentence encoder, we used an embedding set (Wikipedia 2014+Gigaword 5) pretrained by Glove, each of 50 dimensions; for the rest part of the sentence encoder (e.g., position feature and CNN structure), we followed the parameters reported in Zeng et al. (2014). Other parameters were tuned on the validation set. We called stochastic gradient decent to optimize the model, and grid search was harnessed to find the optimal parameters (underscored)—initial learning rate λ∈{0.01, 0.1, 0.3, 0.5}, and learning rate decay γ∈{0.01, 0.1, 0.3, 0.5}. That is, λ is multiplied by γ every *s* training steps. We ran the model on the validation set with *s* = 5, 000, every 2, 000 iterations for 8 epoches, and the best epoch was chosen for testing. In testing, 3, 000 mini-batches are sampled for models to predict, and the prediction accuracy is used as the evaluation metric.

#### 5.1.3. Experiment Setup

We denote our prototypical networks with attention-based MIL as “AMProto.” The variants with maximum, average, and perceptron pooling are denoted as “Proto+MAX,” “Proto+AVE,” and “Proto+PER,” respectively. The competitors^5^ include SNAIL (Mishra et al., 2018), GNN (Satorras and Estrach, 2018), MAML (Finn et al., 2017) as well as prototypical networks (“Proto”), Proto with self-attention (“Proto+Self”) (Wu et al., 2020), and Proto with hybrid attention (“Proto+HATT”) (Gao et al., 2019). SNAIL tackles few-shot learning by temporal CNNs with attention mechanism; GNN models each support and query instance as a node in a graph to learn from past experience; MAML optimizes parameters by maximizing the sensitivity of the loss functions of new tasks.

The widely-applied *N*-way *K*-shot setting was adopted, *N*∈{5, 10} and *K*∈{5, 10}. We tested all models five times, and the *average* results are reported. For fair comparison, we evaluated all models at *instance bag level* (Jiang et al., 2018), i.e., to predict a relation for an instance bag concerning the same entity pair. For the competing approaches that do not work at instance bag level, e.g., Proto (Gao et al., 2019), we trained them at instance level, and chose the instance with the highest confidence score in the instance bag as query in testing.

### 5.2. Experiment Results

#### 5.2.1. Overall Performance

Table 1 reports the accuracy results of different models. From the results, we would like to highlight that (1) all prototypical networks-based methods exhibit better accuracy than other options (i.e., SNAIL, GNN, and MAML); among the rivals, Proto+HATT is the most competitive since hybrid attention is trained to focus on more important support instances and feature dimensions; (2) AMProto outperforms other prototypical networks-based FSRC models, implying that it is more robust for the task of FSRC under distant supervision, since it introduces attention-based MIL method to solve the false positive query problem; (3) on two datasets, the corresponding accuracy of different models is quite similar, demonstrating that FSRC models perform similarly on recognizing old relations and fresh relations.

**Table 1:** Overall results of models.

**Methods**	**Basic version**				**Alternative version**			
	**5 -way 5 -shot**	**5 -way 10 -shot**	**10-way 5-shot**	**10-way 10-shot**	**5-way 5-shot**	**5-way 10-shot**	**10-way 5-shot**	**10-way 10-shot**
SNAIL	62.46 ± 0.37	68.11 ± 0.28	53.49 ± 0.25	56.22 ± 0.27	61.34 ± 0.35	64.77 ± 0.47	54.35 ± 0.31	57.80 ± 0.36
GNN	63.48 ± 0.59	67.92 ± 0.68	49.07 ± 0.63	54.80 ± 0.61	61.58 ± 0.86	63.28 ± 0.64	50.58 ± 0.74	54.88 ± 0.72
MAML	72.58 ± 0.48	74.46 ± 0.64	56.88 ± 0.41	60.45 ± 0.87	70.37 ± 0.71	73.41 ± 0.56	57.56 ± 0.46	61.97 ± 0.44
Proto	73.03 ± 0.23	75.31 ± 0.18	58.46 ± 0.24	61.86 ± 0.23	71.57 ± 0.32	73.80 ± 0.27	59.55 ± 0.26	62.24 ± 0.21
Proto+Self	73.14 ± 0.31	75.55 ± 0.33	58.51 ± 0.26	62.04 ± 0.25	72.24 ± 0.33	74.63 ± 0.34	59.41 ± 0.28	62.79 ± 0.26
Proto+HATT	73.51 ± 0.11	76.96 ± 0.18	58.85 ± 0.15	63.79 ± 0.17	73.17 ± 0.12	76.60 ± 0.22	59.89 ± 0.12	63.42 ± 0.16
AMProto	74.58 ± 0.21	78.38 ± 0.19	61.51 ± 0.22	65.58 ± 0.18	75.23 ± 0.25	77.86 ± 0.22	62.13 ± 0.17	65.41 ± 0.15

#### 5.2.2. Comparison of Different MIL Methods

**Figure 5:**
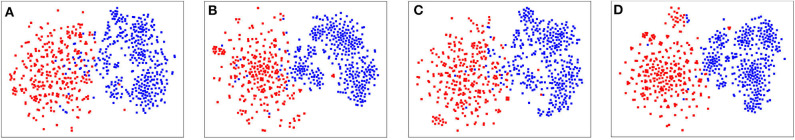
PCA analysis of instance bag embeddings. **(A)** Proto+MAX, **(B)** Proto+AVE, **(C)** Proto+PER, **(D)** AMProto.

To verify the effectiveness of the attention-based MIL, we proceed with comparison analysis by replacing the attention-based MIL with other MIL methods, including MAX, AVE and PER. In this set of experiments, for all models, we keep sampling instance bags as queries in training and testing. The embeddings of these query bags are projected into 2D points by using Principal Component Analysis (PCA), which are shown in Figure 5.

The accuracy results are enumerated in Table 2. From the results, it reads that (1) the attention-based MIL outperforms all other MIL methods, since self-attention allows the high-quality instances in query bag to guide better instance selection. Besides, due to the interaction between query set and support set, a more informative query feature vector contributes to a more representative prototype. (2) Three competing MIL methods achieve similar performance since they all fail to consider information to guide the assignment of weights over multiple instance in the query bag. The noise contained in query bag also misleads the selection of support instances to form the prototype.

**Table 2:** Comparison of different MIL methods.

**Methods**	**Basic version**				**Alternative version**			
	**5-way 5-shot**	**5-way 10-shot**	**10-way 5-shot**	**10-way 10-shot**	**5-way 5-shot**	**5-way 10-shot**	**10-way 5-shot**	**10-way 10-shot**
Proto+MAX	73.48 ± 0.18	76.33 ± 0.14	59.02 ± 0.16	62.24 ± 0.12	73.94 ± 0.17	74.57 ± 0.11	61.19 ± 015	62.82 ± 0.09
Proto+AVE	73.45 ± 0.23	76.52 ± 0.23	59.88 ± 0.19	63.53 ± 0.18	73.89 ± 0.07	75.74 ± 0.12	61.50 ± 0.12	63.25 ± 0.14
Proto+PER	73.17 ± 0.07	77.02 ± 0.15	60.38 ± 0.22	63.63 ± 0.11	73.59 ± 0.13	75.83 ± 0.05	60.66 ± 0.09	63.04 ± 0.05
AMProto	74.58 ± 0.21	78.38 ± 0.19	61.51 ± 0.22	65.58 ± 0.18	75.23 ± 0.25	77.86 ± 0.22	62.13 ± 0.17	65.41 ± 0.15

#### 5.2.3. PCA Projection Analysis

This experiment helps appreciate the predictive effect of different MIL methods visually. We conjecture that Proto+MAX, Proto+AVE, and Proto+PER underperform AMProto, due to the selection of high-quality instances in the query bag and the representation of query feature vector. To validate, we randomly selected 400 query instance bags of two arbitrary relations, and encoded them with different models.

(1) there is a subtle difference in the distribution of feature vectors by Proto+MAX, Proto+AVE, and Proto+PER; and (2) in contrast, feature vectors by AMProto are apt to be linearly-separable when dual attention is exerted; (3) the comparison between AMProto and other MIL methods indicates that the proposed attention-based MIL can learn more distinguishable representation for query bags.

#### 5.2.4. Case Study

We look into the case that AMProto predicts correctly but others fail, to qualitatively show the effectiveness of attention-based MIL. Table 3 presents a sampled case, where both support instances and query bags are selected from the experiment under 5-way 5-shot setting. Particularly, we presented all instances in support set, and the query instance bag which contains four sentences concerning the entity pair (*bobcat, lynx*). The proposed AMProto extracts the relation parent_taxon between *bobcat* and *lynx* based on all sentences of the instance bag. In this way, a triplet (*bobcat*, parent_taxon, *lynx*) can be formed to complete existing knowledge bases.

**Table 3:** Sample instances in case study.

**Relation**: **parent_taxon Entity pair in query**: **(bobcat, lynx)**		**Attention score**
	① it concludes that the closest living link to the galapagos tortoise, or $\underline{\text{geochelone}}$ nigra, is probably a relatively small $\underline{\text{tortoise}}$ found in South America.	0.16
Support instances	② botanically, the poinsettia is $\underline{\text{euphorbia}}$ pulcherrima, a member of the $\underline{\text{euphorbiacae}}$ family, a spurge that comprises about 5,000 specie.	0.27
	③ other examples of convergence include $\underline{\text{marsupial}}$ mammals related to kangaroos and $\underline{\text{opossums}}$ that evolved into creatures resembling lions and wolves.	0.23
	④ a show-stopper was the $\underline{\text{capra}}$ pizza, in which the zing of $\underline{\text{goat}}$ cheese played off beau- tifully against red and yellow bell pepper slices, black olives and a touch of sage.	0.09
	⑤ by a fluke of nature, a $\underline{\text{wildcat}}$ species—$\underline{\text{felis}}$ silvestris tartessia—has survived un- changed for the past 20,000 years in the mountains of Spain.	0.25
	① bobcats or $\underline{\text{bobcat}}$ tracks have been sighted in the hudson river palisades region, but the $\underline{\text{lynx}}$ rarely ventures south of Northern New England and New York state.	0.18
Query instance bag	② the $\underline{\text{lynx}}$ and the $\underline{\text{bobcat}}$ are similar in size and appearance, although the former's ear tufts are more prominent and its feet larger.	0.28
	③ through a complicated chain of events, it was the $\underline{\text{bobcat}}$ that drove the $\underline{\text{lynx}}$ from New York in the late 1800's.	0.23
	④… they may have been inspired by the wide footprints of the snowshoe hare and the $\underline{\text{lynx}}$ (a mountain version of the $\underline{\text{bobcat}}$ with especially large feet) in flight.	0.31

*The mention in blue and underline is the head entity, while the mention in red and underline is the tail entity*.

It can be seen that our self-attention pooling over the query bag can find the common semantic relation expressed by and distinguish the instances of high attention scores that well express the parent_taxon relation, from those of low scores that are mislabeled. Besides, given the query bag, our model can find the high-quality support instances, and assign the lowest attention score to the fourth instance which describes the target relation implicitly.

#### 5.2.5. Results on FewRel Dataset

The two versions of test sets of DS-Few are constructed automatically by distant supervision. To show the performance of few-shot relation classification models on the human labeled data, we also tested our proposed AMProto and all competing methods on FewRel dataset. Specifically, we used the train set of DS-Few to train these models, and the best epochs on the validation set are picked for testing. In our experiments, we tested all few-shot relation classification models on the public train set of FewRel which contains 64 relations. We tested all models on the train set of FewRel due to two reasons: (1) the test set of FewRel is not publicly available; (2) the train set of FewRel contains more relations than the test set (containing 20 relations), which is more challenging for few-shot relation classification models. The results are listed in Table 4. From the results, we can read that our proposed AMProto still achieves the best performance among all models when they are tested on the human labeled data.

**Table 4:** Results on FewRel.

**Methods**	**5-way 5-shot**	**5-way 10-shot**	**10-way 5-shot**	**10-way 10-shot**
SNAIL	61.49 ± 0.31	66.43 ± 0.28	54.32 ± 0.36	52.48 ± 0.43
GNN	64.73 ± 0.45	66.62 ± 0.34	50.65 ± 0.37	53.53 ± 0.51
MAML	70.38 ± 0.25	74.52 ± 0.32	60.49 ± 0.24	62.46 ± 0.33
Proto	71.42 ± 0.52	75.44 ± 0.19	61.35 ± 0.28	63.21 ± 0.48
Proto+Self	72.24 ± 0.62	76.39 ± 0.26	62.75 ± 0.22	64.66 ± 0.27
Proto+HATT	72.78 ± 0.24	76.78 ± 0.27	62.47 ± 0.34	65.52 ± 0.26
AMProto	73.85 ± 0.64	77.32 ± 0.36	63.68 ± 0.46	66.27 ± 0.35

#### 5.2.6. Manual Evaluation

When we use the extracted triplets to boost the knowledge base, we usually select those with high confidence scores. It is because we should guarantee the quality of the triplets. Therefore, the precision of top-*k* triplets (i.e., *P@k*) is an import metric to evaluate few-shot relation classification models. Specifically, we ranked all extracted triplets according to their confidence scores and calculated the precisions at top-*k* triplets. In our experiments, we tested all models under 5-way 5-shot and 5-way 10-shot settings on the basic version of test set. Table 5 presents the precisions at top-100, top-200, and top-300. It reads from the results that our proposed AMProto outperforms all baselines. Therefore, it is safer to employ AMProto than other models to boost knowledge base.

**Table 5:** The *P@k* values.

**Methods**	**5-way 5-shot**				**5-way 10-shot**			
	***P*@100**	***P*@200**	***P*@300**	**Average**	***P*@100**	***P*@200**	***P*@300**	**Average**
SNAIL	85.00	83.00	79.33	82.44	86.00	84.00	80.67	83.56
GNN	83.00	81.50	79.67	81.39	85.00	85.50	80.33	83.61
MAML	90.00	89.50	86.33	88.61	93.00	91.00	87.67	90.56
Proto	92.00	90.50	87.67	90.06	94.00	91.50	88.67	91.39
Proto+Self	94.00	92.00	88.33	91.44	96.00	93.50	89.67	93.06
Proto+HATT	94.00	92.50	89.33	91.94	97.00	94.00	89.67	93.56
AMProto	95.00	94.00	90.67	93.22	98.00	95.50	91.33	94.94

## 6. Conclusion

In this paper, to union the advantages of distant supervision and few-shot learning, we have investigated the task of few-shot relation classification under distant supervision. To evaluate, we reconstruct existing distant supervision data to confront the scenario of FSRC. Seeing the unique challenges, we conceive a attention-based multiple instance learning method over prototypical networks to mitigate the mislabeled instances in both support set and query set. Other multiple instance learning approaches, including maximum pooling, average pooling, and perceptron pooling, are selected as our baselines. Empirical study verifies the feasibility of the task and the superiority of the method over other few-shot learning models and various baselines. From the experimental results, we can see that our proposal is more robust to the challenging task.

Our research is evaluated under the classic *N*-way *K*-shot setting of few-shot learning, which can be applied into the scenario of extracting triplets from free texts in designed blanks of forms. However, the real-world application is more complicated. Specially, more free texts may express no relation or other relation not in the support set, which cannot be handled by our proposed model and the competing methods. In the future, we will extend our work to solve the problems of negative instances and cross-domain texts, and enable it to be applicable to more complicated scenario of relation classification.

## Data Availability Statement

The raw data supporting the conclusions of this article will be made available by the authors, without undue reservation.

## Author Contributions

NP wrote the paper and conducted the experiments. WX instructed the experiments. HX prepared the data. ZT revised the manuscript. All authors contributed to the article and approved the submitted version.

## Conflict of Interest

The authors declare that the research was conducted in the absence of any commercial or financial relationships that could be construed as a potential conflict of interest.

## Footnotes

^1^A download link to the data will be provided in the final version.

^2^https://github.com/thunlp/PathNRE

^3^In this study, clusters with <15 instance bags were discarded, because in the 10-shot settings, at least 15 unique instance bags are necessary—10 as support instance bags and 5 as queries.

^4^For ease of comparison, FewRel is of 100 relations and 70,000 instances.

^5^We omitted comparing with LM-ProtoNet (FGF) (Fan et al., 2019), as (1) all model parameters were unreported, in the original paper, and (2) it performs not as good as Proto+HATT (Gao et al., 2019).
